# Longitudinal Melanonychia in Children: Clinical and Histopathologic Features and Management with Literature Update

**DOI:** 10.3390/dermatopathology13010013

**Published:** 2026-03-23

**Authors:** Isabelle Moulonguet, Marie Caucanas, Sophie Goettmann

**Affiliations:** 1 Dermatopathology, Cochin Hospital, 27 Boulevard Saint Jacques, 75014 Paris, France; 2 Dermatology, 132 Bis Boulevard Pierre et Marie Curie, 31200 Toulouse, France; marie_caucanas@hotmail.com; 3 Dermatology, 4 Rue du Maréchal Harispe, 75007 Paris, France; sophie.nail@gmail.com

**Keywords:** longitudinal melanonychia, pediatric longitudinal melanonychia, subungual melanocytic proliferation, subungual nevus, nail unit

## Abstract

Longitudinal melanonychia (LM) results from the deposition of pigment in the nail plate due to increased melanocytic activity within the nail matrix. Recent publications on this topic have helped clarify the main clinical, histological, and evolutionary characteristics of pediatric LM and provide guidance for its appropriate management. In this review, we will examine the literature on the subject. LM is far less common in children than in adults and is most often caused by benign nail matrix lesions. Pediatric LM has specific clinical and histopathologic features, and many of the clinical warning signs used in adults are not applicable to children. Pediatric lesions may show atypical cytologic and even architectural features yet still follow a benign clinical course. Spontaneous regression of LM is common in children, with fading and narrowing of the pigmented band, or even complete disappearance. The vast majority of pediatric LM cases can be managed conservatively with regular follow-up, including clinical photography and onychoscopy.

## Simple Summary

Longitudinal melanonychia, defined as a pigmented band along the nail plate, is uncommon in children and is most often caused by benign melanocytic lesions of the nail matrix, particularly nevi. However, these lesions frequently display clinical, dermoscopic, and histopathologic features that may resemble melanoma in adults, creating diagnostic uncertainty. Nail unit melanoma in children is extremely rare, and its diagnosis remains controversial in the literature. Because many warning signs used in adults are not reliable in pediatric patients, management strategies must differ. Most cases can be safely managed with careful clinical and dermoscopic follow-up rather than immediate biopsy. Surgical intervention should be reserved for lesions showing significant clinical concern, as nail procedures in children are technically challenging and may lead to permanent nail dystrophy. A cautious and conservative approach, ideally involving clinicians and dermatopathologists experienced in nail disorders, is therefore recommended when evaluating longitudinal melanonychia in children.

## 1. Introduction

Longitudinal melanonychia (LM) is a band-like pigmentation that originates in the nail matrix and extends distally along the nail plate. It results from the deposition of pigment within the nail plate due to increased melanocytic activity in the nail matrix.

LM is far less common in children than in adults, and its underlying causes differ from those observed in adults. Recent publications on this topic have helped clarify the main clinical, histological, and evolutionary characteristics of pediatric LM and provide guidance for its appropriate management. In children, LM most commonly results from benign nail matrix nevi (NMN). However, the clinical presentation of these lesions may be atypical and could raise concerns of malignancy if observed in adult patients. Furthermore, even benign pigmented lesions of the nail matrix may show atypical histopathologic features when biopsied.

Consequently, the diagnostic approach to pediatric LM—including the decision to perform a biopsy—requires different considerations from those applied in adults. Subungual melanoma is exceptionally rare in childhood, and its diagnosis is often controversial.

Appropriate management of longitudinal melanonychia in children therefore requires knowledge of its specific clinical and histologic characteristics. The present article aims to analyze the clinical and histopathologic features of melanocytic nail lesions in childhood. Because understanding the histologic features of the normal nail apparatus is essential for evaluating melanocytic lesions, this review will also summarize these characteristics.

## 2. Histologic Features of the Normal Nail Apparatus

### 2.1. Proximal Nail Fold and Lateral Nail Folds

The dorsal portion of the proximal nail fold and the lateral nail folds resemble the skin of the dorsal surface of the finger. In contrast, the ventral surface of the proximal nail fold (the eponychium) is characterized by a thinner and flatter epidermis and lacks sweat glands.

**Figure 1:**
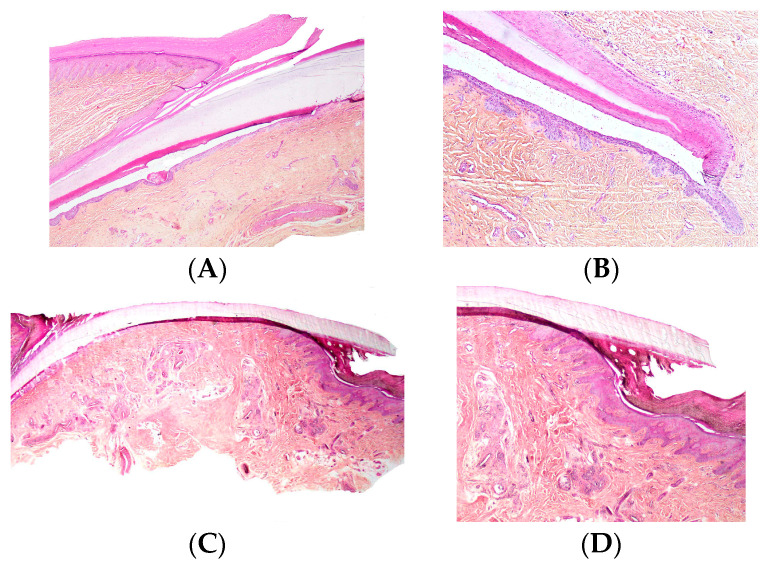
Longitudinal section of nail unit. (**A**) Proximal nail unit. The proximal nail fold, including the cuticle, and the nail matrix are seen. The proximal nail fold is similar to the epidermis of the proximal digit, though it lacks hair follicles. (**B**) Nail matrix. (**C**) Section from the cuticle to the distal edge of the nail plate. (**D**) Distal edge of nail plate, showing the hyponychium subjacent to the free, distal edge of the nail plate.

At the junction between the dorsal and ventral layers, the proximal nail fold produces a thick keratinized layer known as the cuticle. The cuticle functions as a seal that protects the nail apparatus by preventing microorganisms from penetrating beneath the proximal nail fold (Figure 1A). The integrity of the proximal nail fold is therefore essential for maintaining nail health.

### 2.2. Nail Matrix

The nail matrix extends from the proximal nail fold to the distal part of the lunula (Figure 1B). The proximal matrix produces most of the nail plate (approximately 80–90%), forming its dorsal portion, whereas the distal matrix generates the remaining ventral or deep part.

The matrix consists of several layers of basophilic cells with sparse cytoplasm and no granular layer. Beneath this lies a prekeratogenous layer composed of polygonal cells with abundant eosinophilic cytoplasm, oval nuclei, and fine chromatin, followed by a keratogenous zone. The keratogenous zone is characterized by elongated cells with eosinophilic cytoplasm that are incorporated into the surface of the nail plate. The prekeratogenous and keratogenous zones together form the onychogenic band. Distally, the keratogenous zone gradually becomes thinner and ultimately disappears at the transition between the nail matrix and the nail bed [1].

The proximal matrix corresponds to the point at which the nail epithelium reflects onto the ventral surface of the proximal nail fold. In longitudinal section, it appears as a bulbous structure of variable size located between the epithelium of the ventral proximal nail fold and the distal matrix. In the distal matrix, the matrix epithelium is thinner and the onychogenic zone smaller, giving it a flatter appearance. In the central part of the nail, the matrix is thick and displays oblique ridges that extend posteriorly, whereas papillomatosis is less pronounced laterally.

The matrix continues distally into the nail bed. Its dermis consists of a thin papillary dermis and a thicker reticular dermis containing few adipocytes and lacking a true hypodermis.

### 2.3. Nail Bed and Isthmus

The transition between the nail matrix and the nail bed is marked by the disappearance of the onychogenic band. The nail bed epithelium consists of a single basal cell layer and a well-developed spinous layer but lacks a keratogenous zone. In transverse sections, papillomatosis is clearly visible and oriented parallel to the longitudinal axis of the nail, whereas in longitudinal sections the nail bed epithelium appears relatively flat (Figure 1C).

The isthmus represents a transitional zone between the distal nail bed and the hyponychium and contributes to sealing the subungual region. In this area, the epithelium is thicker, and keratinization produces a parakeratotic band that firmly adheres to the distal subungual surface [1,2]. This band is composed of elongated cells with clear cytoplasm and flattened nuclei.

The granular layer reappears at the hyponychium, which represents the thick distal portion of the nail bed and is bordered by the distal nail groove and the fingertip (Figure 1D). In the nail bed, the dermis appears homogeneous and, as in the matrix, there is no true hypodermis.

### 2.4. Nail Plate

The nail plate is produced by the nail matrix. It is composed of parallel layers of flattened cells known as onychocytes. These cells adhere tightly to one another and, unlike corneocytes of the epidermis, do not undergo desquamation. Residual nuclei are often present in the ventral portion of the nail plate. In the pathology laboratory, the nail plate is extremely hard and therefore difficult to section [1]. Several agents can be used to soften the plate, including Mollifex Gurr (VWR Int. Ltd., Radnor, PA, USA), 10% potassium hydroxide solution, or 10% potassium thioglycolate cream [3]. Other methods include immersing the paraffin block in hot water for 5–10 min or applying a few drops of 10% potassium hydroxide to the paraffin block before sectioning [1,3].

### 2.5. Melanocytes

#### Distribution and Activity

The number of melanocytes in the nail apparatus is considerably lower than in the epidermis of the skin, with approximately 217 melanocytes/mm^2^ compared with about 1150 melanocytes/mm^2^ in the epidermis [4]. Melanocytes in the nail apparatus also differ in their usual quiescence. In the proximal matrix, most melanocytes are dormant and do not produce pigment. In the distal matrix, approximately 50% are dormant and 50% are active [4]. This difference in activity explains why LM more frequently originates in the distal nail matrix. In the nail bed, melanocytes are even rarer, ranging from absent to approximately 50 melanocytes/mm^2^, and they are typically dormant [4]. The presence of suprabasal melanocytes is physiological in the matrix. In the proximal matrix, melanocytes are usually located within the second to fourth germinative layers, whereas in the distal matrix they are found in the first and second basal layers [4]. Normal melanocyte nuclei are small and oval and are similar in size to—or slightly larger than—adjacent keratinocyte nuclei. They are also slightly more hyperchromatic [1].

### 2.6. Melanocyte Density and Immunohistochemistry

1. Melanocyte density is typically assessed by counting the number of intraepithelial melanocytes per millimeter along the epithelial–dermal junction of the nail matrix or nail bed. In the normal adult nail matrix, the density ranges from approximately four to nine melanocytes per millimeter (mean: 7.7/mm) [5]. Melanocytes are small and often possess fine dendritic processes.
2. Immunohistochemical staining using nuclear melanocytic markers such as Sox10 or MITF is preferred for assessing melanocyte density. These markers facilitate accurate visualization of melanocytes while avoiding pitfalls such as excessive dendritic staining that may occur with markers such as Melan-A or HMB-45. S100 protein has been shown to be unreliable in this setting because it may fail to label matrix melanocytes [6]. Consequently, S100 should not be used alone for this purpose. PRAME appears to be a relatively sensitive and specific marker for distinguishing benign from malignant melanocytic lesions of the nail in adults. However, its interpretation must always be correlated with morphological findings [7,8]. To date, no studies have evaluated PRAME in children, and adult nail-unit data cannot be directly extrapolated to the pediatric population. Ultimately, the diagnosis of melanocytic nail lesions should rely on a combination of clinical, histologic, and immunohistochemical features.

## 3. Nail Samples: Surgical Techniques and Specimen Handling

Biopsy for the diagnosis of longitudinal melanonychia (LM) requires adequate sampling of the nail matrix, which is protected by the nail plate and the proximal nail fold [9]. Partial sampling may lead to delayed or missed diagnosis. The most accurate diagnosis requires examination of the entire lesion through an excisional biopsy [10].

The choice of surgical procedure depends on several factors, including the location of the band (median or lateral), its width, and the presumed origin within the nail matrix [10].

For pigmented bands less than 3 mm wide originating from the distal matrix, a 3 mm punch biopsy is generally appropriate. Latero-longitudinal excision is preferred for lateral pigmented bands [10].

Bands wider than 3 mm or those originating from the proximal matrix pose a greater challenge because of the risk of permanent nail dystrophy [9]. These lesions may be removed by elliptical excision or by tangential matrix excision (also referred to as nail matrix shave biopsy). This technique, described by Haneke, allows tangential removal of a melanocytic focus from the matrix with limited postoperative dystrophy, even when large areas are excised [9,11,12,13]. The procedure removes the matrix epithelium along with a thin layer of the underlying dermis [9,11,12,13].

Proper specimen handling is essential for accurate histopathologic evaluation. If the specimen is placed freely in fixative, it may shrink and lose its orientation [14]. To prevent this, the specimen can be placed on filter paper that is folded over to enclose it [15]. Alternatively, the specimen may be positioned on cardboard containing a nail diagram and covered with filter paper or a sponge before being placed in a tissue cassette [13]. The cassette is then immersed in fixative.

The pathology laboratory must ensure correct orientation of the specimen before paraffin embedding [10]. Punch biopsies measuring 3–4 mm are usually embedded intact without sectioning. If histologic examination shows that the sections are properly oriented, serial sections are prepared. Once the lesion is adequately represented on the slides, deeper sections and immunohistochemical studies can be performed. The paraffin block should be properly trimmed to ensure that the lesion is accurately represented. If the block is not sufficiently sectioned, the proliferative area may be poorly visible on the slides, potentially leading to a misdiagnosis.

The gold standard for diagnosing pigmented nail unit lesions remains sampling of the nail matrix. However, recent studies have shown that histopathologic examination of nail clippings may provide valuable information in the evaluation of melanonychia in adults [16,17,18,19,20,21,22]. Because melanocytes from the nail matrix epithelium may become incorporated into the nail plate, melanocyte remnants can sometimes be identified during histopathologic examination of the nail plate [16,17].

Several studies have suggested that the presence of melanocytic remnants in nail plate clippings may represent a clue to underlying nail unit melanoma in adults [16,17,18,19,20,21,22]. However, melanocytes may also be present within the nail plate in cases of nail matrix nevi in children. Therefore, interpretation of nail plate findings must always take the patient’s age into account [19,20,21,22].

## 4. Longitudinal Melanonychia in Children

### 4.1. Pathophysiology

Longitudinal melanonychia results from the deposition of melanin in the nail plate. This occurs either through increased melanin production by melanocytes (melanocytic activation) or through an increased number of melanocytes (melanocytic proliferation).

When nail matrix melanocytes are stimulated, melanosomes are transferred through dendritic processes to the surrounding epithelial matrix cells. These cells then migrate distally and differentiate into onychocytes that form the nail plate, producing a linear pigmented band [10]. In melanocytic proliferations, the increased number of melanocytes results in greater melanin production and consequent nail pigmentation [11].

### 4.2. Clinical Data on LM in Children

The median age of onset of childhood LM is between four and six years, while the first medical consultation typically occurs around eight years of age [23,24,25]. In a recent meta-analysis of 24 studies including 1391 pediatric patients, the most frequent sites were fingernails (76.2%), most commonly affecting the first digits (thumb or hallux) (45.4%) [26]. The relative incidence of LM differs markedly between children and adults. In adults, melanocytic activation represents the most common cause of LM. In contrast, in children the most frequent cause is benign melanocytic proliferation, including nevus or lentigo. A study of 40 children under 16 years of age with LM found that nevi (47.5%) and lentigines (30%) accounted for 77.5% of cases [27]. Another series analyzing 100 biopsies from children with LM identified 22 nail matrix nevi [28]. In 12 patients, the band was present at birth or had first been noted during early childhood; in the remaining cases, it appeared after puberty. In a recent meta-analysis including 1391 pediatric patients, nevus was the most frequent cause of LM (86.3%), followed by lentigo (3.0%) and melanocytic activation (1.3%) [26].

Free-edge nail plate onychoscopy can help localize the lesion within the nail matrix. In most cases, pigmentation is visible on the ventral nail plate, indicating a distal matrix origin [29].

**Figure 2:**
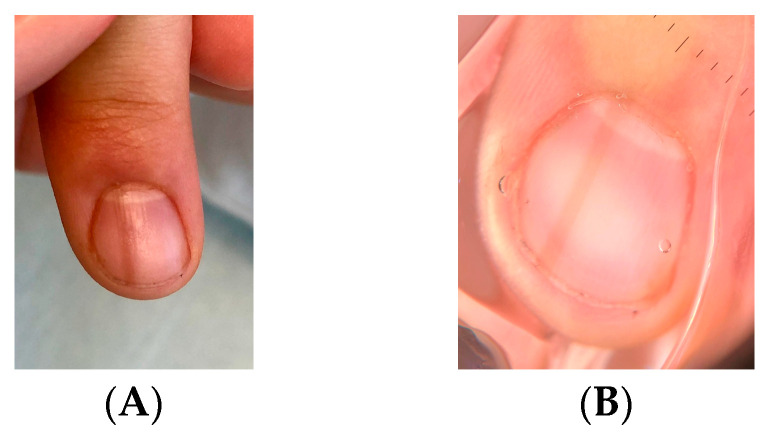
(**A**) Broad, homogeneous, light-brown LM in a 12-year-old girl. (**B**) Onychoscopy: thin band of homogeneous color.

**Figure 3:**
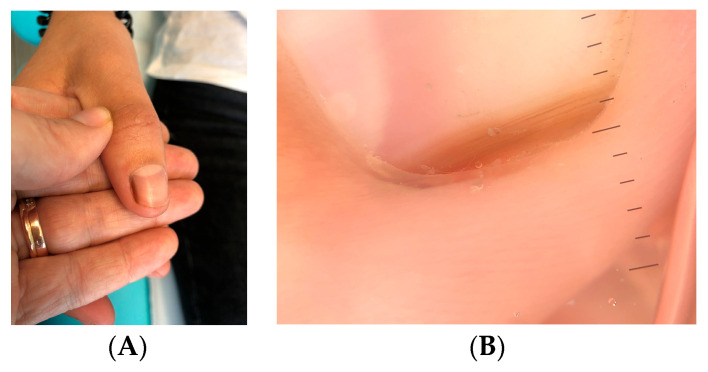
(**A**) Brown LM of the right thumb in a 15-year-old girl. (**B**) Onychoscopy: thin, more or less pigmented bands with break in continuity and with blurred edges.

**Figure 4:**
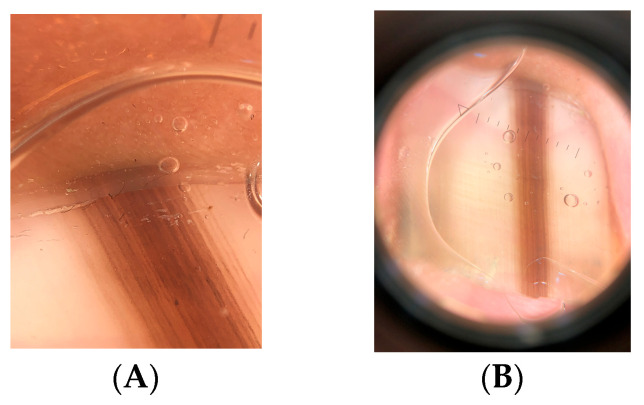
LM in a 15-year-old boy. (**A**,**B**) Onychoscopy: brown background with brown lines that are irregularly spaced, with varying widths.

**Figure 5:**
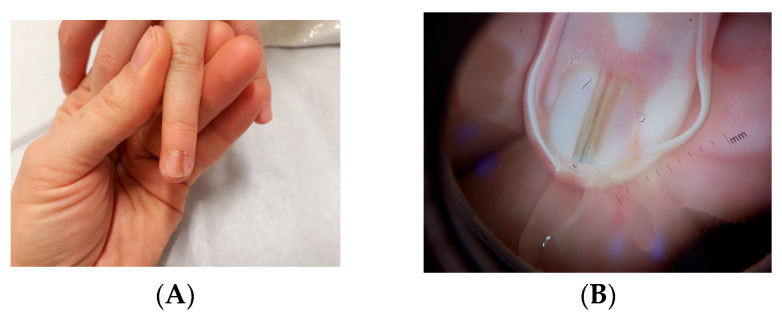
(**A**) Irregular LM of the left ring finger in a five-year-old boy. (**B**) Onychoscopy: streaks of unequal size and color, disruption of continuity and loss of parallelism.

**Figure 6:**
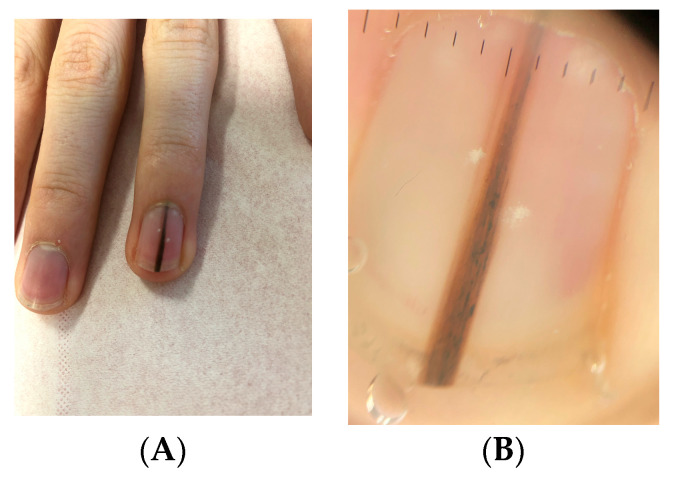
LM of the ring finger in a 15-year-old boy. (**A**) Dark, triangular-shaped LM (narrower base) indicating a progressive nature. (**B**) Onychoscopy: presence of dots and lines indicate regression.

**Figure 7:**
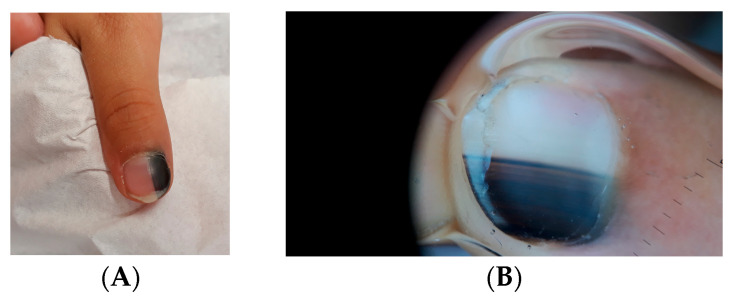
(**A**) Dark LM with pseudo-Hutchinson sign in a five-year-old boy. (**B**) Onychoscopic appearance which allows clear visualization of the presence of very dark parallel bands, of irregular widths, and the pseudo-Hutchinson sign.

**Figure 8:**
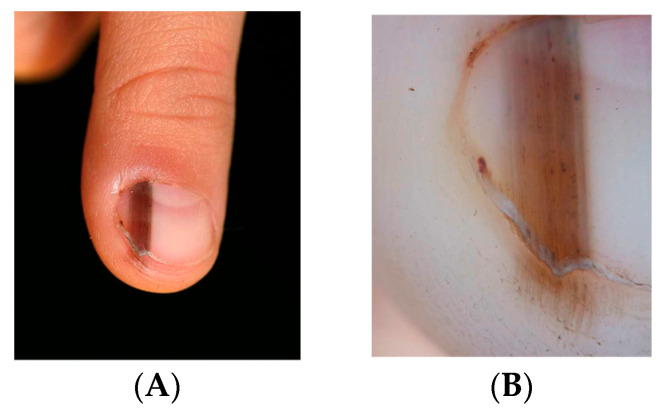
(**A**) LM of the thumb of a seven-year-old child. (**B**) Onychoscopic appearance of longitudinal parallel streaks of variable color and thickness with dots and lines. Note the brush-like appearance on the hyponychium which is different from the haphazard pigmentation of a Hutchinson sign.

Clinically, LM in children often appears as a single, narrow, homogeneously pigmented band that is well demarcated from the adjacent normal nail plate (Figure 2A). The typical dermoscopic feature of nail matrix nevus is the presence of regular parallel lines on a brown background (Figure 2B). However, pediatric LM may present with atypical clinical and dermoscopic features (Figure 3, Figure 4, Figure 5, Figure 6, Figure 7 and Figure 8). In some cases, these features would strongly suggest nail melanoma if observed in adults, making interpretation challenging [23,24,25,26,29,30,31,32,33,34,35,36,37,38,39,40,41,42,43,44,45,46,47,48,49,50]. In a meta-analysis, Tsai found that a high proportion of benign pediatric LM displayed atypical features, including: dark-colored bands (69.8%), multicolored bands (47.6%), broad band width (41.1%), irregular patterns (38.1%), nail dystrophy (18.2%), and triangular signs (10.9%) [26]. The triangular sign refers to a band that is broader proximally than distally, suggesting lesion growth. A pseudo-Hutchinson sign corresponds to pigmentation of the nail matrix visible through the cuticle and was observed in 41% of cases. A true Hutchinson sign, caused by extension of the melanocytic proliferation into the periungual skin, was reported in 23.7% of cases. A micro-Hutchinson sign refers to periungual pigmentation visible only under dermoscopy. Because of these findings, diagnostic criteria used for subungual melanoma in adults are not directly applicable to children. Changes in width or color are frequently reported at the time of presentation, partly because evolving lesions are often referred for biopsy.

In a multicenter international prospective study of 230 children younger than five years with congenital or congenital-type LM, Pham et al. described their main clinical and dermoscopic characteristics [50]. They distinguished congenital nevi present at birth from congenital-type nevi acquired between birth and five years of age. A substantial proportion of these lesions displayed worrisome clinical and dermoscopic features, including: broad pigmented bands, nail dystrophy, triangular shape, polychromia, micro-Hutchinson sign, blurred borders, and irregular longitudinal microlines. In 27.8% of cases, a distinctive distal fibrillar (“brush-like”) dermoscopic pattern was observed, which appears to be characteristic of pediatric nevi [41,50]. Congenital nail matrix nevi more frequently exhibited periungual pigmentation, Hutchinson sign, and the distal fibrillar pattern than congenital-type nevi [51]. Apart from these differences, both groups shared similar initial characteristics [50]. Unlike in situ melanoma, none of the congenital or congenital-type nevi demonstrated a parallel ridge pattern or a diffuse irregular pattern in the periungual skin. A peculiar zigzag dermoscopic pattern has also been described in childhood and early adulthood LM, almost exclusively affecting the thumb. It appears as a brown band composed of spiral or zigzag shapes [49,52,53].

Melanocytic activation represents the third cause of LM in children and is more often observed in patients with darker skin phototypes. It results from increased melanin production by a normal number of activated matrix melanocytes [29]. In children, melanocytic activation is often secondary to nail-biting (onychophagia) or nail-picking (onychotillomania) [54]. Drug-induced melanonychia and systemic syndromes such as Peutz–Jeghers syndrome or Laugier–Hunziker syndrome are rare in childhood.

Dermoscopy is widely used to help differentiate these etiologies. In general, the color of the background and the lines may indicate whether the lesion results from melanocytic activation (gray coloration) or proliferation (brown-black pigmentation) [55]. Nevertheless, although clinical and dermoscopic features are helpful, histologic examination remains necessary for definitive diagnosis.

### 4.3. Evolution of Pediatric LM

**Figure 9:**
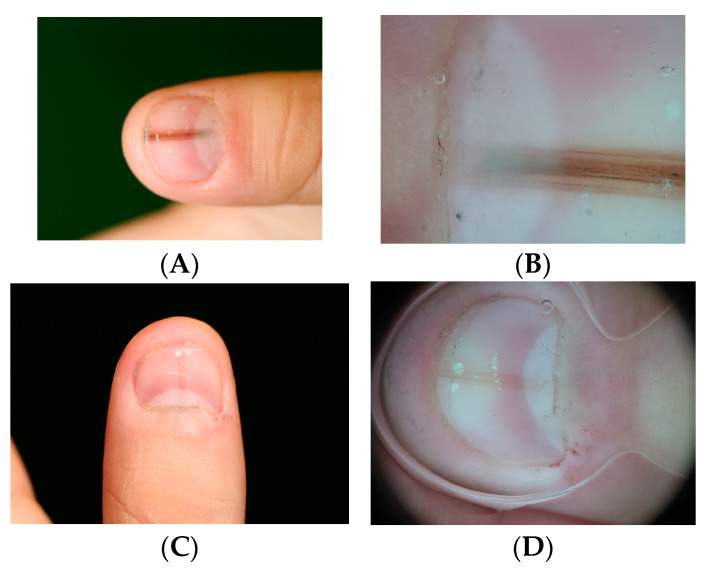
(**A**,**B**) LM of the thumb with longitudinal streaks irregular in color and width with loss of parallelism, evolving towards regression. (**C**,**D**) After three years, regression with disappearance of the LM. The figures were obtained from Prof. Luc Thomas of Hospital Lyon Sud France.

Several studies have investigated the natural course of LM in children. These lesions often initially show rapid enlargement and darkening, followed by stabilization and, in many cases, partial or complete regression during adolescence [23,24,25,30,34,43,47,48]. Spontaneous regression is common and may manifest as progressive fading or narrowing of the pigmented band, or even complete disappearance [56,57,58] (Figure 9). In Tsai’s meta-analysis, most lesions either remained stable (23.3%) or underwent spontaneous regression (19.9%) during follow-up [26]. In a study of 60 patients younger than 16 years evaluating changes in LM width, progression stopped in 97.5% of cases, and 50% of lesions began to regress after a median of 29.5 months [25]. Another retrospective study including 381 pediatric patients found that 48.5% showed no change in color or width, 15% initially darkened, and 16% initially widened before stabilizing [23]. Lightening or narrowing occurred in 42% of cases, and 5.5% completely regressed during follow-up, with a median time to complete regression of three years [23]. Single lesions located on the left hand or foot and showing homogeneous coloration were positively associated with complete regression [23]. Hutchinson sign partially or completely regressed in 50% of cases; in half of these cases, it initially worsened before regressing [23].

In Tsai’s meta-analysis, 23.3% of lesions remained stable, 19.9% regressed spontaneously, and 29.9% continued to evolve with changes in width or color over time [26]. Recurrence of the melanocytic proliferation after biopsy or surgical excision may occur [32,36,37,38]. These recurrences likely reflect the poor circumscription of these lesions as well as the technical difficulty of achieving wide margins while preserving the function and cosmetic appearance of the digits [32].

When atypical lesions are partially biopsied, complete excision is generally recommended to minimize recurrence and the potential risk of aggressive behavior [32].

### 4.4. Histologic Features of LM

#### 4.4.1. Melanocytic Activation

In melanocytic activation, there is no increase in melanocyte density. Histologically, only a few melanocytes with pigmented dendritic processes and pigmented keratinocytes are observed. In some cases, the pigmentation is sparse and may require Fontana–Masson staining to be identified. This stain may also highlight occasional thin melanocytic dendrites. A small number of melanophages are frequently present in the superficial dermis. Immunohistochemical staining with melanocytic markers confirms that the number of melanocytes is normal and that no melanocytic proliferation is present. Melanocytic activation can sometimes be difficult to distinguish from lentigo, which is characterized by a slight increase in melanocyte density. In such cases, pathologists can identify melanocytic activation but may not be able to determine its underlying cause.

#### 4.4.2. Melanocytic Proliferation

Melanocytic proliferation is defined as an increase in the number of melanocytes within the nail matrix. It may correspond to a lentigo when melanocytes are isolated or to a nevus when at least one melanocytic nest (theca) is present.

**Figure 10:**
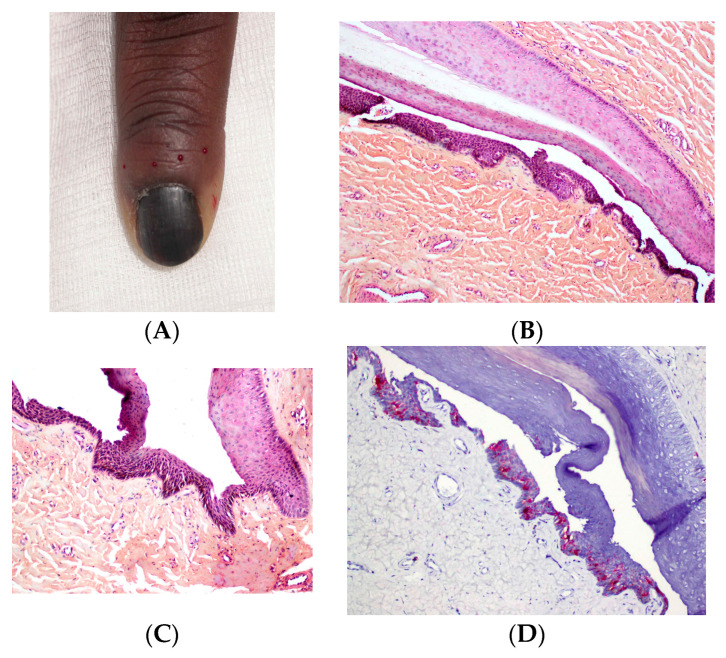
Lentigo. (**A**) Total melanonychia in a 10-year-old child. (**B**,**C**) Histopathology (HES), proliferation of isolated cells. (**D**) MelanA immunostaining showing positive isolated cells.

Lentigo is characterized by a mild to moderate increase in the number of matrix melanocytes (Figure 10). These melanocytes usually remain isolated without confluence. Elongation of epidermal ridges is generally much less pronounced than in cutaneous lentigo. Cytologic atypia is mild or absent. Occasional focal areas of pagetoid migration may be observed but typically remain limited. Pigmentation is often confined to the lower third of the epithelium, although it may sometimes extend more extensively. The superficial dermis frequently contains a small number of melanophages. In some cases, the degree of melanocytic hyperplasia is subtle, making differentiation from melanocytic activation difficult.

**Figure 11:**
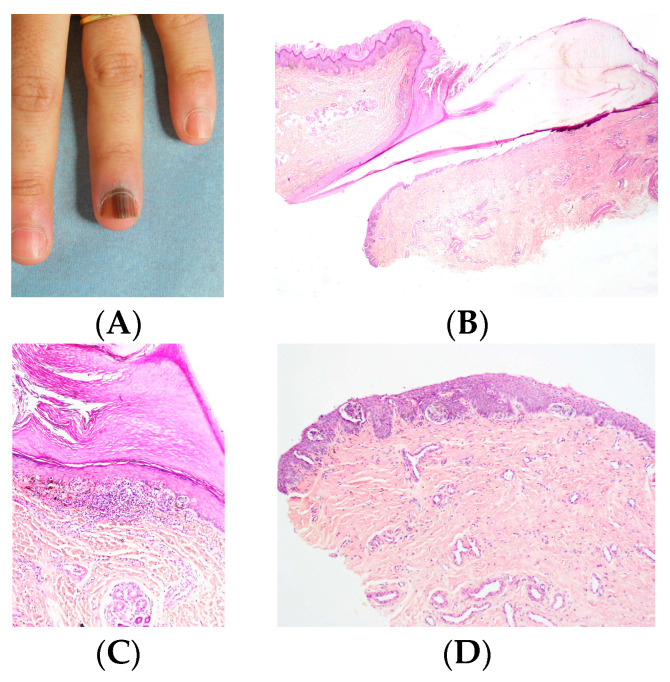
Junctional nevus. (**A**) LM in a 10-year-old child, bands of irregular color with pseudo-Hutchinson sign. (**B**) The proliferation is located in the ventral part of the proximal fold and in the matrix (HES). (**C**) Melanocytic nests in the proximal nail fold (HES). (**D**) Melanocytic nests in the matrix (HES).

In nevi, melanocytic nests (thecae) are present within the matrix. In some cases, these nests may also be located in the ventral portion of the proximal nail fold and/or the hyponychium, which may explain the periungual pigmentation frequently observed in pediatric nail nevi (Figure 11).

**Figure 12:**
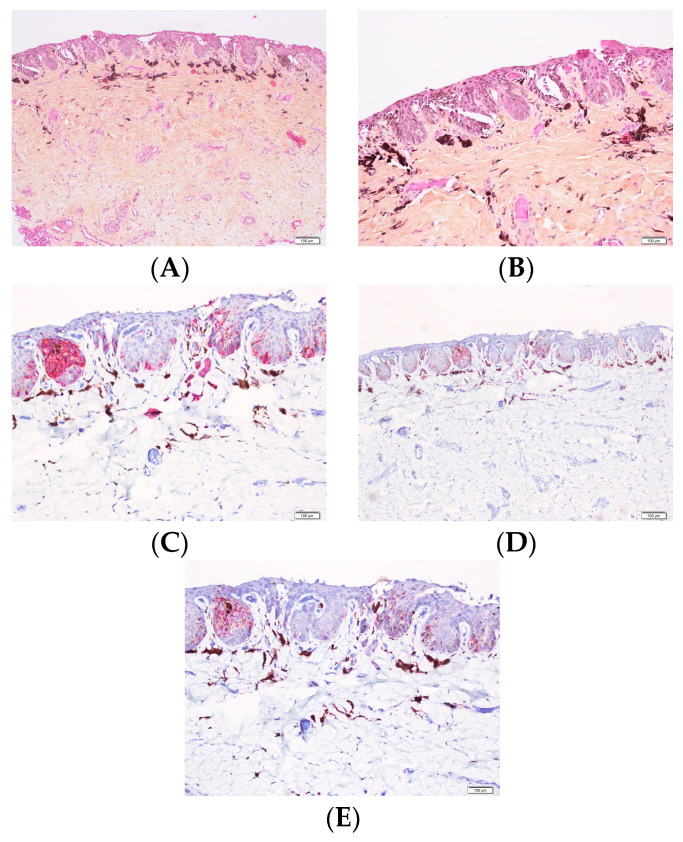
Junctional nevus in a 12-year-old child. (**A**,**B**) Melanocytic nests in the matrix (HES). (**C**) Melan-A stain. (**D**) Sox10 stain. (**E**) PRAME stain.

**Figure 13:**
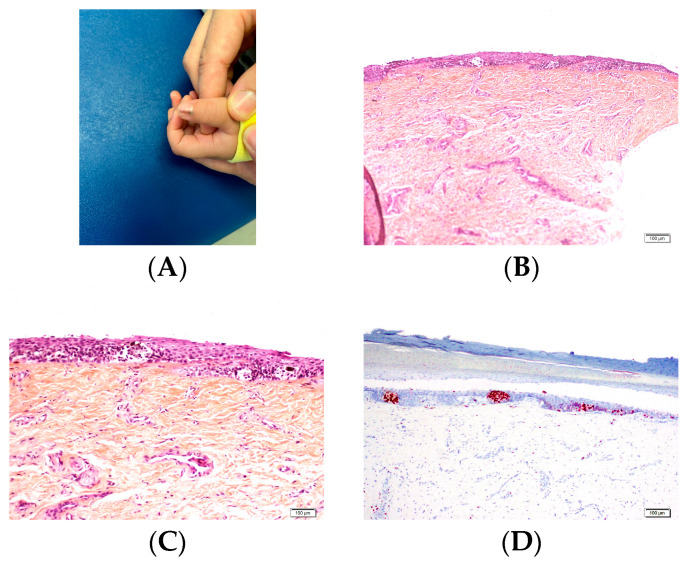
Junctional nevus. (**A**) LM in a seven-year-old boy with irregular LM and pseudo-Hutchinson sign. (**B**,**C**) Proliferation of melanocyte nests with some isolated cells (HES). (**D**) Sox10 stain showing the nests.

**Figure 14:**
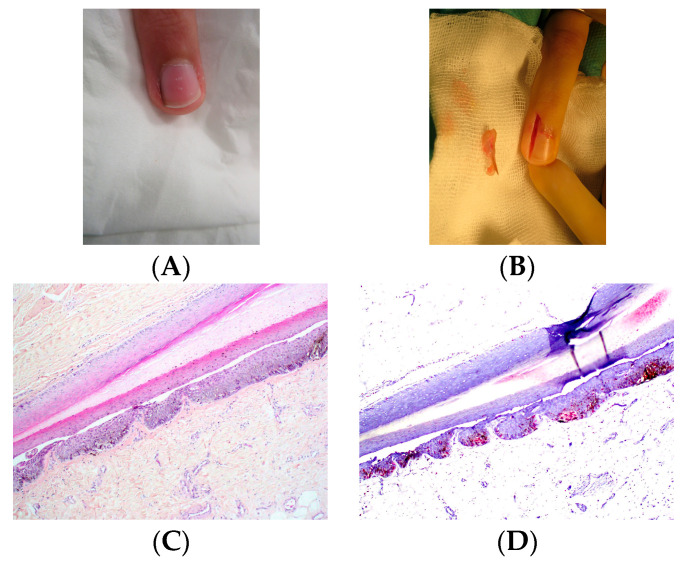
Junctional nevus. (**A**) LM in a 10-year-old child. (**B**) Longitudinal excision of the melanonychia. (**C**) Histopathology (HES). Matrix proliferation consisting of regular thecae leading to the diagnosis of nevus. (**D**) Sox10, labeling of melanocyte nuclei.

Most nevi of the nail apparatus are junctional. They often exhibit a predominantly nested growth pattern (Figure 12, Figure 13 and Figure 14). The dominant cytomorphology consists of small round to epithelioid melanocytes, although dendritic or epithelioid morphologies may also predominate.

**Figure 15:**
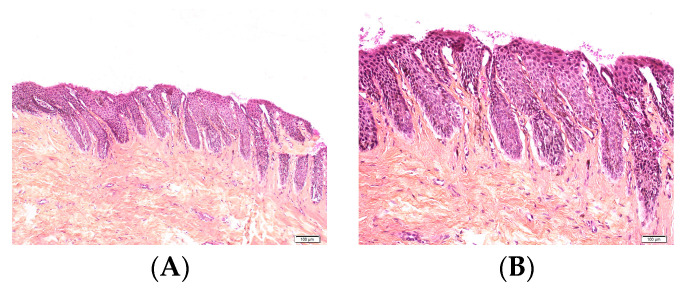

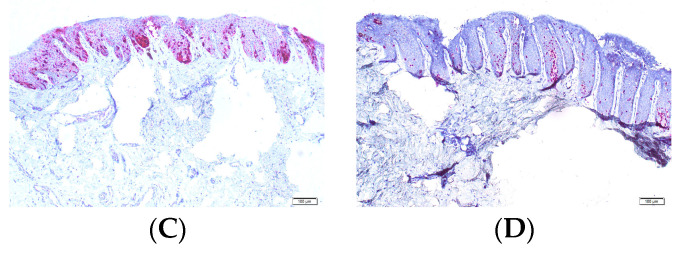
Junctional nevus. (**A**,**B**) Matrix proliferation with nests and numerous isolated cells (HES). (**C**) Melan-A stain. (**D**) Sox10 stain.

**Figure 16:**
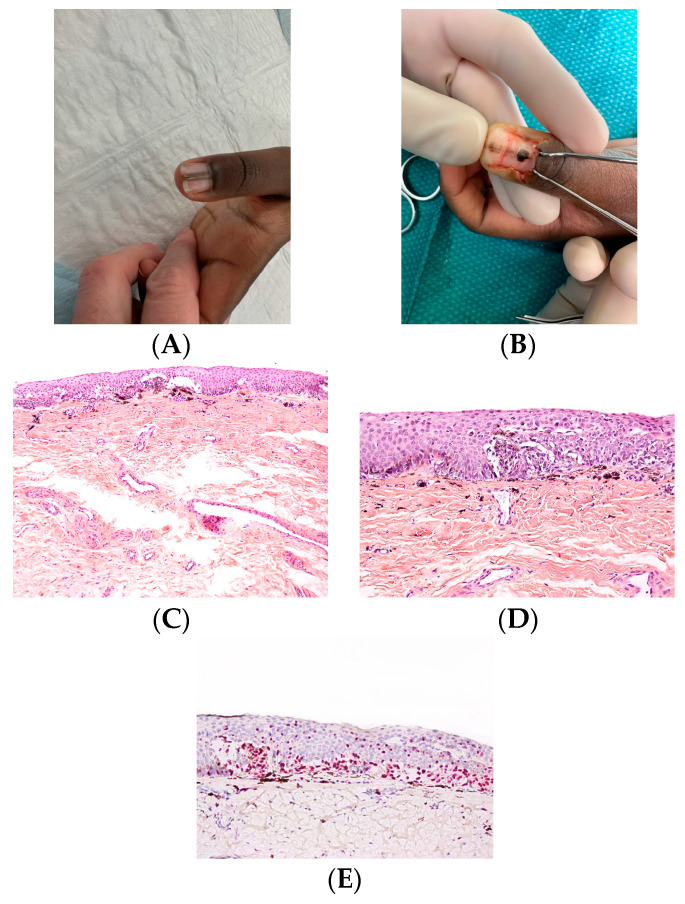
Junctional nevus. (**A**) Dark LM in a 14-year-old girl with nail dystrophy. (**B**) Excision of the black pigmentation of the matrix. (**C**,**D**) Proliferation of nests with some isolated cells (HES). (**E**) Sox10 stain highlighting the nests and the isolated melanocytes.

**Figure 17:**
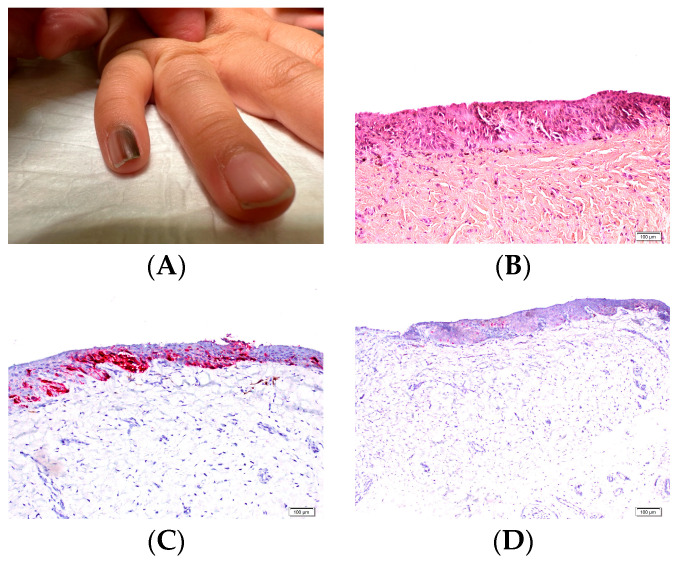
Junctional nevus. (**A**) Large and irregular LM in a 12-year-old child. Coll Dr A Salon. (**B**) Proliferation of isolated cells with few nests (HES). (**C**) Melan-A stain. (**D**) PRAME stain.

In some cases, lentiginous melanocytes predominate over junctional nests (Figure 15, Figure 16 and Figure 17). Variable nuclear atypia, including nuclear enlargement, hyperchromasia, or nuclear angulation, may be observed. In adult patients, such features would raise concern for melanoma [32,36,37,38].

However, only a few cases of pediatric nail unit melanoma presenting as LM have been reported in the literature [32,33,59,60,61,62,63,64,65,66,67,68,69]. The diagnostic accuracy of these cases has been questioned because of their indolent clinical behavior [29,30,31,32,33]. Since melanocytic proliferations in children may display worrisome clinical and histopathologic features, and because no definitive uniform histopathologic criteria exist, the diagnosis of melanoma in children should be approached with great caution.

In pediatric LM, melanocytes may sometimes be very numerous, with melanocyte counts (MC)—defined as the number of melanocytes per millimeter along the subungual dermoepithelial junction—exceeding 36 [36,37,38]. For comparison, in a study by Amin, the MC in subungual melanoma in situ averaged 58.9, with a range of 39 to 136 [6]. Data on melanocyte counts in the pediatric nail matrix are scarce, which complicates the assessment of cytologic changes such as atypia or proliferation [29]. Ren proposed a threshold MC of 40 to define atypical melanocytic hyperplasia [38]. However, the melanocyte count must always be integrated with the overall architectural pattern (lentiginous versus nested), the extent and distribution of suprabasal spread, cytologic atypia, clinicodermoscopic evolution, and longitudinal follow-up.

Suprabasal spread of melanocytes or even extensive upward migration into the upper epidermis is uncommon but may occasionally be encountered. The terms *atypical junctional melanocytic hyperplasia* or *atypical lentiginous melanocytic proliferation* may be used to describe such lesions [32,36,37,38]. Histopathologic features—including variable nuclear atypia and increased numbers of single melanocytes with suprabasal or pagetoid spread—share many similarities with previously reported cases interpreted as pediatric nail unit melanoma in situ [32,36,37,38].

Significant interobserver variability among pathologists may occur, reflecting the differences in histologic patterns of atypia in pediatric versus adult nail melanocytic lesions. Difficult cases should therefore be evaluated by dermatopathologists with specific expertise in nail pathology.

When a diagnosis of subungual atypical lentiginous melanocytic proliferation is made on biopsy or excision, fluorescence in situ hybridization (FISH) may help with risk stratification. However, experience with FISH in pediatric nail melanocytic lesions remains limited. In pediatric LM, FISH has been reported in only two series totaling 17 patients [32,38]. Among these cases, gain of 11q13 (CCND1) was identified in one patient, whereas the remaining 16 showed no copy number changes [32,38]. Due to the limited available data, FISH results should be interpreted cautiously in pediatric cases [33].

**Figure 18:**
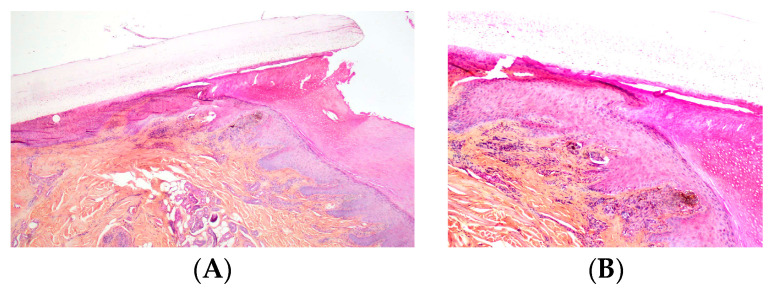
Compound nevus in a 12-year-old girl. (**A**,**B**) Proliferation of junctional and dermal nests at the hyponychium.

Only three cases of ungual Spitz nevus have been reported to date in dermatologic journals [70,71,72]. One of these cases presented clinically with a zigzag pattern [70]. Histopathologic examination demonstrated a symmetrical and well-circumscribed proliferation of spindle and epithelioid melanocytes located at the dermoepidermal junction and forming vertical melanocytic nests. However, junctional nevi of the nail apparatus often display a similar pattern of vertical nests composed of pigmented epithelioid cells. Therefore, the designation of Spitz nevus for these lesions remains controversial, particularly because other characteristic features of Spitz nevi are lacking and the published illustrations are limited. In compound nevi, dermal nests are most often observed in the hyponychium (Figure 18).

### 4.5. Management of Longitudinal Melanonychia in Children

Recommendations on the management of LM in children are difficult to formulate. A major challenge in evaluating pediatric LM is the absence of a clearly defined age threshold for applying pediatric versus adult diagnostic criteria [29,33,46]. The onset of puberty may represent a potential transition point, but data on this issue remain limited. Another unresolved question concerns the natural evolution of LM from childhood into adulthood and whether its risk profile differs from that of LM arising de novo in adults.

As discussed previously, pediatric subungual melanocytic lesions have specific clinical and histologic characteristics, and many of the warning signs used in adult melanonychia are not applicable in children. Consequently, pediatric LM should be managed differently from adult LM. Most authors agree that the vast majority of pediatric LM cases can be managed conservatively, thereby avoiding the risk of unnecessary invasive procedures. Clinical follow-up with digital dermoscopic imaging is appropriate in most situations. When reassuring clinical and dermoscopic features are present, biopsy can generally be avoided. Even in lesions with concerning features, regular clinical monitoring over an extended period—typically every 3–6 months until stability is confirmed—is often recommended. Many benign pediatric LM lesions either stabilize or undergo spontaneous regression. Nail unit biopsy should be reserved for lesions displaying high-risk clinical features. Performing nail unit biopsies in children is challenging because of both physical and psychological distress and because of the risk of permanent nail dystrophy.

Histologic diagnosis may also be difficult when biopsy samples are small, increasing the risk of incomplete excision, persistence, or recurrence of the lesion. When surgery is required, complete excision is preferable whenever possible, as it allows more reliable histologic assessment and reduces the risk of recurrence.

Another challenge concerns the choice of surgical approach. In adults, melanocytic lesions are usually confined to the nail matrix, and excision of this area is generally sufficient to establish the diagnosis and remove the lesion. In children, however, nevi may extend beyond the matrix to involve the proximal nail fold or the hyponychium [33]. Consequently, the surgical approach should be carefully discussed among the family, dermatologist, and surgeon.

When the child is older and able to tolerate local anesthesia—often after adolescence—surgical excision may be considered, particularly in cases of total melanonychia, in order to avoid lifelong clinical monitoring.

## 5. Limitations

Interpretation of current data on pediatric longitudinal melanonychia is limited by several key factors. A primary challenge is the absence of a clearly established age threshold differentiating pediatric from adult diagnostic criteria. Puberty is sometimes proposed as a transition point, but its timing varies considerably among individuals, leaving a peripubertal/adolescent “grey zone” in which the biological behavior of nail melanocytic lesions is uncertain. Practically, clinicians should individualize management in this group: younger children can generally be managed conservatively with clinical and dermoscopic follow-up, whereas older adolescents nearing or completing puberty may be assessed using adult criteria, particularly if the lesion demonstrates high-risk features or evolution over time.

Additional limitations include selection bias, as most published data derive from referral-center cohorts or retrospective case series, overrepresenting atypical or worrisome lesions. The true incidence of pediatric nail unit melanoma is extremely low, further constraining the ability to define reliable clinical, dermoscopic, or histopathologic criteria. Considerable interobserver variability among pathologists may occur when interpreting pediatric nail melanocytic lesions, reflecting differences in histologic features compared with adults. Finally, the diagnostic utility of immunohistochemical markers and ancillary molecular tests, including fluorescence in situ hybridization (FISH), is limited in pediatric cases due to scarce validation and uncertain interpretive significance. Collectively, these factors underscore the need for cautious interpretation of suspicious lesions, individualized decision-making, and multicenter prospective studies to better guide management in children and adolescents.

## 6. Conclusions

Longitudinal melanonychia is far less common in children than in adults, with a median age of onset between four and six years and a predominance of lesions affecting the fingernails.

In most cases, pediatric LM is caused by a nail matrix nevus. These lesions may exhibit clinical, dermoscopic, and histologic features that would be considered alarming in adults. Nevertheless, the vast majority are benign. Nail unit melanoma in children is exceptionally rare, and its diagnosis remains controversial.

In most cases, a conservative “watch-and-wait” approach with regular clinical and dermoscopic follow-up is the most appropriate management strategy. Nail unit surgery should be reserved for lesions with high-risk clinical features, given the potential complications of nail procedures, particularly permanent nail dystrophy.

Difficult cases should be evaluated by dermatopathologists with specific expertise in nail pathology, as significant interobserver variability may occur in the interpretation of pediatric nail melanocytic lesions.

## Data Availability

No new data were created or analyzed in this study.
